# Evaluation of a COVID-influenced Curriculum to Address Food Insecurity in a Detroit Family Medicine Residency Clinic

**DOI:** 10.51894/001c.17649

**Published:** 2020-10-30

**Authors:** Amrien Ghouse, William Gunther, Matthew Sebastian

**Affiliations:** 1 Integrative Medicine Northwestern University; 2 Palliative Care University of Michigan; 3 Family Medicine Beaumont Farmington Hills

**Keywords:** diabetes mellitus, nutrition education, primary care, food insecurity, hemoglobin a1c

## Abstract

**CONTEXT:**

To date, numerous projects have demonstrated that an ongoing limited access to nutritionally dense food (i.e., “food insecurity”) plays a key role in the overall health and wellbeing of lower income at-risk populations.

**METHODS:**

For this 2019-2020 pilot project, the resident physician authors first created and administered a simple five-item questionnaire screening process to systematically identify food insecure patients in their metropolitan Detroit residency clinic. A sample of patients who had been identified as food insecure and pre-diabetic were then provided improved access to healthy foods, supplemented by a six-week program of nutritional education classes using a nationally recognized “Cooking Matters’’ six-week long curriculum with a licensed chef and nutrition educator

**RESULTS:**

After institutional review board approval, the authors enrolled a sample of 10 adults. The authors successfully measured both pre- and post-program Hemoglobin A1c (HbA1C) levels for all participants who completed the required course and subsequent clinic follow up visits. Using a series of initial non-parametric Wilcoxon Signed Rank matched pair tests, post-program follow-up at three months revealed statistically significant reductions in documented HbA1c levels from baseline for six enrolled patients (W=1, Z = - 2.226, p = 0.026) and six-month follow up (i.e., more than four months after completion of the program) (W = 1, Z = - 2.060, p = 0.039). In post-program surveys, each respondent indicated that they found the class content to be generally beneficial to increase their nutritional knowledge.

**CONCLUSIONS:**

In the authors’ setting, this food insecurity program has subsequently led to a more formal screening process to evaluate and identify food insecure patients. The authors discuss the scheduling difficulties they experienced from the COVID-19 pandemic for their sample patients. However, these pilot results suggest that prolonged benefits may require ongoing “virtual” teaching sessions with pre-diabetic patients to address the complex factors influencing food insecurity levels identified in similar inner-city settings.

## INTRODUCTION

A growing body of evidence has identified the association between dietary factors and mortality in patients with Coronary Artery Disease,^1^ Cerebrovascular Accidents (CVA),^2,3^ Hypertension,^4,5^ Type 2 Diabetes Mellitus,^6,7^ and cardio-metabolic disease.^1–8^ The nutritional factors associated with these kinds of adverse health outcomes include high intake of high sodium processed meats, sugar sweetened beverages, and lower intake of fruits, vegetables, nuts/seeds, and omega 3 fats.^1^

Estimated proportional diet-related cardio metabolic mortality (i.e., relationship between certain diet related factors and cardiovascular related death) has been found to be higher among men, individuals with low or medium education levels, and African American and Latino populations.^9–11^ Several additional factors influencing consumption of suboptimal dietary components as identified by clinic-based providers include perceived time constraints, food taste, impaired access to more nutritional foods and cost.^12,13^

During project planning, the authors concluded with colleagues that many patients at their Michigan primary care residency clinic were consuming poorer-quality diets consisting of high-fat calorie laden foods with low nutrient density. During their scheduled pre-project clinic office visits, physicians had tried to routinely provide patients with basic dietary and exercise advice and brief recommendations to implement “diet and lifestyle changes” into their lifestyles as recommended in the 2015 United States Preventative Service Task Force Guidelines.^14^

Although this practice was found to be somewhat effective for those of perceived middle/upper class status, the authors found patient adherence with dietary and exercise recommendations was often compounded by unreported lower-income patients’ socioeconomic factors not readily shared with providers. Patients would often not share that issues related to transportation, lack of income, or insecure housing as reasons for not adhering to their medical plan of care.

“Food insecurity” has been characterized as a phenomenon of limited access to nutritionally dense foods with ample access to “unhealthy” food options.^15^ Before the project, the residents had anecdotally concluded that many of their pre-diabetic clinic patients were likely food insecure and unable to implement changes due to multiple complex factors placing them at higher risk for developing chronic diseases and need to later initiate diabetes medications.^12^

Even though managing medically complex patients with limited resources is a professional responsibility at our nation’s residency clinics, providers typically lack enough resources (e.g., time, nutritional expertise) and training to identify this vulnerable population.^16,17^ The research to date has also indicated that healthcare service costs (e.g., hospital admission and emergency department visit rates) can be significantly reduced when intensive diet education is provided for higher-risk patients.^16,18,19^

**Figure 1.**
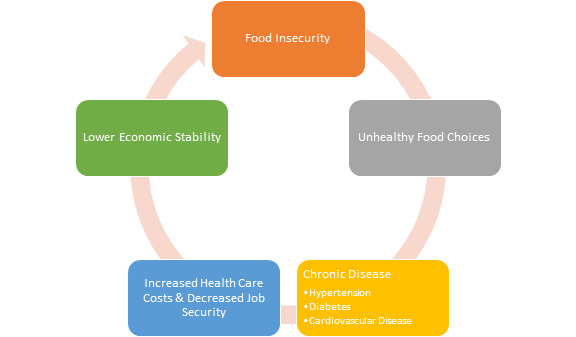
Anticipated Relationship between Food Insecurity and Chronic Disease** (figure created by authors)

Before the project, the 2014 “Map the Meal Gap” Study conducted by Feeding America had confirmed that 3.5% of the population in Oakland County (i.e., 164,499 individuals) and 22.4% of the population in Wayne County (i.e., 394,590 individuals) were currently food insecure.^20,21^ As primary care physicians, the authors and others have believed that food insecurity screening could be feasibly incorporated into preventative health maintenance office visits to improve patients’ chronic disease risks.^17,22^ (Figure 1 created by authors)

### Purpose of Pilot Project

The primary objective of this pilot project was to evaluate the feasibility of a program to identify and address food insecurity among the authors’ pre-diabetic residency clinic population. By providing their food insecure patients with a series of nutrition educational classes and samples of suggested nutritious foods, the authors aimed to primarily improve baseline-to-three-month and baseline-to-six -month changes in patients’ Hemoglobin A1c (HbA1c) levels.

## METHODS

Before data collection, this project design was approved by the Beaumont Farmington Hills institutional review board. Eligible patients were first identified using a voluntary five-item food security screening survey by residents when they checked in for their routine primary care visits.

Eligible adult patients included those: a) with a HbA1c in the American Diabetes Association established prediabetic range (5.7 to 6.4 mg./dl.),^23^ b) identified as “food-insecure” through intake survey (to be described), c) willing to attend a scheduled series of six weekly educational classes with distribution of promoted grocery samples, d) willing to make the types of lifestyle changes to be promoted during classes, and e) who used at least a conversant level of English.

Patients were excluded if they were pregnant or breastfeeding, had any upcoming surgeries, had received prior bariatric surgery, took weight loss supplements or were already enrolled in a medical weight loss program to reduce confounding influences on their prospective A1c changes.^23^

**Figure 2.**
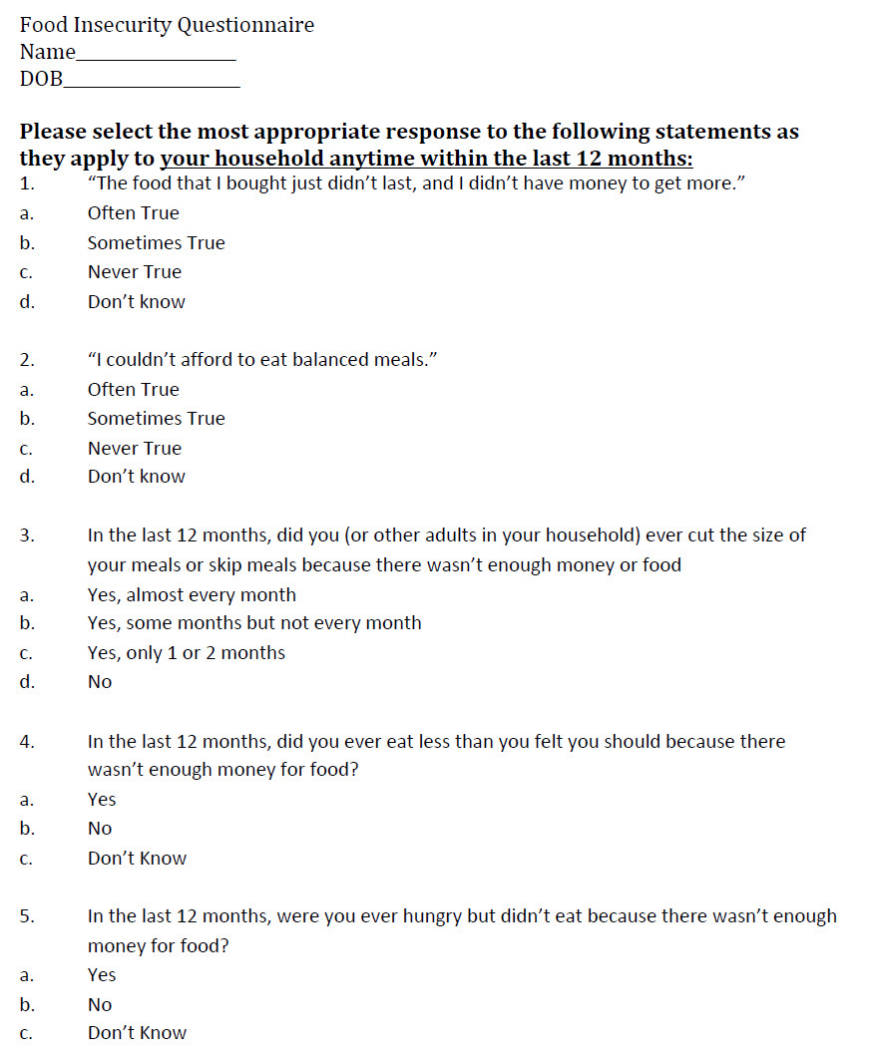
2012 USDA Food Insecurity Questionnaire

Enrolled participants were administered a copy of the United States Department of Agriculture (USDA) five-item food security screening survey upon checking in for their first post-enrollment primary care visit (Figure 2).^24^ This survey and scoring scale had been developed in 2012 at the USDA National Center for Health Statistics and has been widely implemented since 1995.^24^ Survey results were then reviewed by the authors and each eligible patient assigned a composite food security score.

Patients with a HbA1c in the pre-diabetic range and found to have a “low” or “very low” food security score were then invited to be part of the pilot study sample. Eligible patients were required to sign an informed consent to participate in this study. Following the recommendations of class instructors, a maximum of 20 participants were registered for each class and each class was limited to 10 enrolled patients when possible.

The initial pilot sample consisted of ten participants who had enrolled in weekly classes taking place over a period of six weeks led by a licensed chef and nutrition educator. Although the authors were present during each class to assist patients as needed, all classes were led by the certified instructors. The nationally recognized “Cooking Matters” curriculum was taught during each class, focusing on educating people with limited budgets to make healthy affordable food choices.^25^

At each class, participants were provided targeted nutritional education materials, information about household budgeting strategies, shopping tips, and hands on meal preparation techniques. In addition, one session included a healthy grocery store trip where participants were provided a hypothetical budget and tasked with picking healthy options to construct an example meal.

Following each class, a bag of nutritional groceries was provided to each participant to help them implement the techniques they had just learned. Food items included lean chicken breasts, quinoa, fresh vegetables, brown rice, and lentils. Patients who continued to need assistance with food access were provided information concerning a local food pantry that provided no cost food and clothing.

To gauge the anticipated program impact on changes of HbA1c levels from baseline to three-month and six-month follow-up periods, a series of non-parametric (i.e., not based on a normal distribution) Wilcoxon Signed Rank matched pair (i.e., HgA1c of Patient X before classes compared to HbA1c of same patient after classes) t tests^26^ were conducted using *SPSS version 25* analytic software. The authors observed a coefficient alpha of 0.05 to indicate statistical significance.^27^

## RESULTS

Of the ten initially enrolled participants, two patients were unable to attend any scheduled classes citing lack of transportation. One other participant withdrew from the program after attending several classes due to their personal difficulty with the curriculum content, and two participants completed the curriculum classes but did not return for HbA1c lab value rechecks. During subsequent office visits, all three of these patients returned to standard of care diabetic counseling as provided information regarding local food banks.

During later study months, the emergence of the COVID-19 pandemic also seriously prohibited participant attendance as planned classes to bolster our study population had to be cancelled. In the end, approximately 25% of the eligible population were enrolled and completed the series of classes due to this and other logistical limitations (e g., inadequate transportation, poor follow up, childcare obligations).

Program surveys were administered to each enrolled patient by the authors after they had attended at least 50% of scheduled classes, with most mid-program remarks indicating that respondents feeling that the curriculum was helping them increase their nutritional knowledge.

The authors successfully measured both pre and post-program HbA1c levels for over half of participants who completed classes, with each participant experiencing a documented HbA1c reduction. The initial baseline (Mean 6.04 (SD = 0.162, range from 5.80 through 6.30) to three-month (Mean 5.72 (SD = 0.098, range from 5.60 through 5.90) post-program HbA1c reductions for six patients were statistically significant (W = 1, Z = - 2.226, p = 0.026).

**Figure 3.**
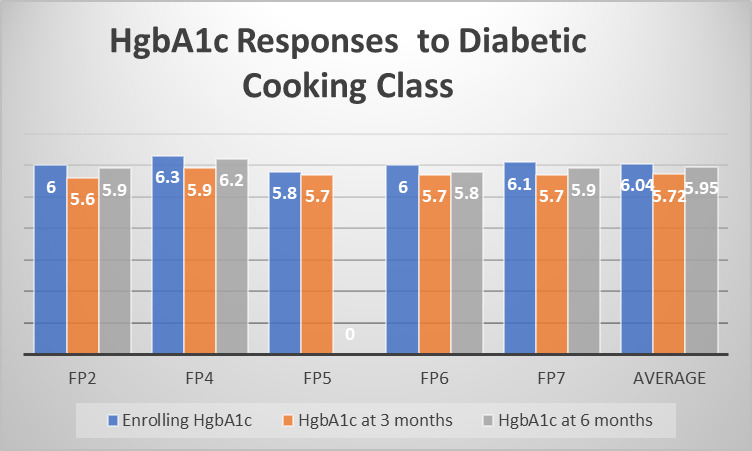
Individual Average HbA1c Reductions after Nutritional # Education Classes (n = 5)

**Figure 4.**
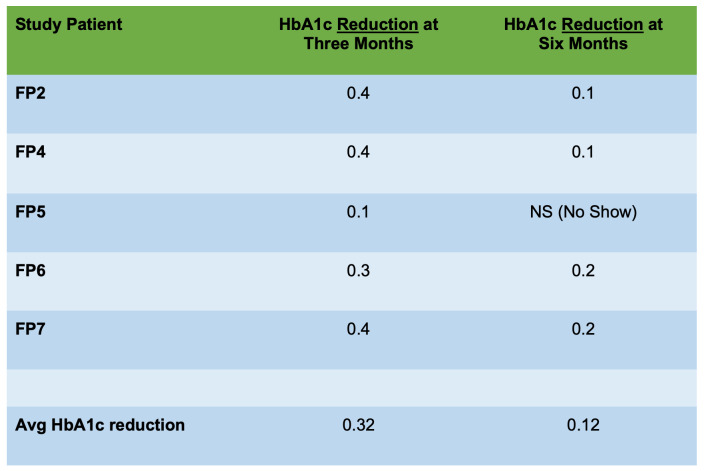
Average HbA1c Post-Program Reductions at Three and Six Months

At six-month follow-up (i.e., more than four months after completion of the program), patients’ subsequent HbA1c reductions were also significant from baseline, decreasing (slightly less) from baseline Mean of 6.04 (SD = 0.125, range from 5.80 through 6.30) to six-month Mean of 5.95 (SD = 0.130, range from 5.90 through 6.20) (W = 1, Z = - 2.060, p = 0.039) for five patients. (Figures 3 and 4)

## DISCUSSION

Food insecurity has been shown to be an important health concern that needs to be addressed during clinic office visits to reduce patients’ onset of several major chronic diseases.^1–8^ Through implementation of this program at our Family Medicine residency clinic, we attempted to foster an educational environment to help reduce the perceived stigma frequently associated with food insecurity and address common barriers to preparing healthy meals (e.g., cost and perceived time constraints).^12^

The perceived benefit of our program was initially evidenced in mid-program survey comments made by participating patients. Patients commented on looking forward to each weekly class to socialize with others facing similar food-related challenges. Each respondent indicated that they had gained valuable nutritional knowledge that they thought they could realistically implement into their daily lives.

Exemplar responses indicated that healthy eating “wasn’t that hard” and “didn’t have to be expensive.” One participant also expressed that it had been difficult “being needy,” and that classes enabled them to avoid ridicule when telling others that they had difficulty obtaining food each week. Being able to participate in this class let them appreciate that they “weren’t alone” and that it was “ok to ask for help.”

Managing lower-income patients experiencing food insecurity is a challenge in most of our nation’s residency clinics.^16,17^ Unfortunately, many patient education programs may fail to adequately identify food insecurity levels among lower income patients.^22^ Patients are frequently forced to make unhealthy food choices due to cost, lack of education or lack of access to nutritional food items.^12,13^ Our demonstrated improvements in HbA1c levels suggest that many pre-diabetic patient’s health can be improved without requiring additional diabetes medications when provided nutritional education and office visit guidance.

## Project Limitations/ Project Impact from COVID-19 Pandemic

Several project limitations include our use of a small convenience sample in a single primary care residency clinic setting. During later recruitment months, our clinical follow-up efforts and educational class attendance rates were also severely restricted by the emerging COVID-19 pandemic, prompting a cessation of our educational classes and inability to recruit more participants. As a result only those in our initial recruitment group were able to complete study classes.

In the future, development on remote “virtual” classes may be beneficial, both due to COVID-19 restrictions and anticipated transportation difficulties in this patient population. An additional follow along cookbook and specific video conferencing instructions could help address potential participation barriers (e.g., harsh weather conditions, unreliable transportation, and social anxiety).

An emerging body of evidence has recently identified disproportionate rates of COVID-19 related hospitalizations, accompanying infections, and mortality for patients already facing health inequities.^28^ Minority group and socially disadvantaged adults who were already facing food insecurity, obesity and obesity-related chronic diseases are now facing staggering COVID-related illness rates.^29^

Although our program participation was associated with measured HbA1c decreases in all sample patients, somewhat smaller decreases from baseline occurred during the post-program months. Future larger-scale studies are needed to more fully investigate the average post-education periods when higher risk patients may begin to lose the benefits from prior classes and when follow up or “refresher” classes would be most impactful. It is imperative to formulate future primary care education and food access/pick up programs for food insecure patients that remain compatible under COVID-19 pandemic circumstances.

## CONCLUSIONS

Food insecurity is an important factor that needs to be identified and addressed during clinic-based office visits to reduce many patients’ chronic disease risks and need for diabetes-related medications. These pilot project results indicate that nutritional education programs may feasibly foster an effective learning environment to reduce the stigma of food insecurity for many of our nation’s pre-diabetic patients and their families.

### Conflicts of Interest

The authors report no conflicts of interest or project funding.
